# Randomized Clinical Trial of the Need for Antibiotic Treatment for Low-Risk Catheter-Related Bloodstream Infection Caused by Coagulase-Negative Staphylococci

**DOI:** 10.3390/antibiotics12050839

**Published:** 2023-05-01

**Authors:** Laia Badia-Cebada, João Carmezim, María-Teresa Pérez-Rodríguez, Elena Bereciartua, Luis-Eduardo López, Marta Represa Montenegro, Virginia Pomar, Marta Andrés, Elizabet Petkova, Nieves Sopena, Jaime Lora-Tamayo, Víctor Monsálvez, Maria Fernanda Ramirez-Hidalgo, Silvia Gómez-Zorrilla, Lucía Boix, Yolanda Meije, Emili Jiménez, Oriol Gasch

**Affiliations:** 1 Internal Medicine Department, Hospital Universitari Parc Taulí, Institut d’investigació i innovació Parc Taulí, Universitat Autònoma de Barcelona, 08208 Sabadell, Spain; 2 Unit of Statistics, Hospital Universitari de Bellvitge/Institut d’Investigació Biomèdica de Bellvitge-IDIBELL, 08908 L’Hospitalet de Llobregat, Spain; 3 Infectious Diseases Unit, Department of Internal Medicine. Hospital Álvaro Cunqueiro, Galicia Sur Health Research Institute, 36312 Vigo, Spain; 4 Infectious Diseases Unit, Hospital Universitario de Cruces, 48903 Barakaldo, Spain; 5 Infectious Diseases and Microbiology Clinical Unit, University Hospital Virgen Macarena, 41009 Seville, Spain; 6 Departament of Medicine, School of Medicine, University of Sevilla, 41009 Seville, Spain; 7 Biomedicine Institute of Sevilla (IBiS)/CSIC, 41009 Seville, Spain; 8 Center for Biomedical Research in Infectious Diseases Network (CIBERINFEC), Instituto de Salud Carlos III, 28029 Madrid, Spain; 9 Infectious Diseases Unit, Department of Internal Medicine, Hospital de la Santa Creu I Sant Pau, 08025 Barcelona, Spain; 10 Infectious Diseases Unit, Department of Internal Medicine, Hospital Consorci de Terrassa, 08227 Terrassa, Spain; 11 Infectious Diseases Unit, Department of Internal Medicine, Hospital Universitario Fundación Jiménez Díaz, 28040 Madrid, Spain; 12 Infectious Diseases Department Hospital Germans Trias i Pujol, 08916 Badalona, Spain; 13 Department of Internal Medicine, Hospital Universitario 12 de Octubre, Instituto de Investigación “imas12” Hospital 12 de Octubre, 28041 Madrid, Spain; 14 Infectious Diseases Department, Hospital Universitari Parc Taulí. Institut d’investigació i Innovació Parc Taulí, Universitat Autònoma de Barcelona, 08193 Sabadell, Spain; 15 Nosocomial Infections Departmen, Arnau de Vilanova University Hospital, 08202 Lleida, Spain; 16 Infectious Diseases Department. Hospital del Mar, Fundació Institut Mar d’Investigacions Mèdiques, Universitat Pompeu Fabra, 08003 Barcelona, Spain; 17 Infectious Diseases Department, Hospital Universitari Mútua Terrassa, 08221 Terrassa, Spain; 18 Faculty of Medicine, Infectious Diseases, Universitat Internacional de Catalunya, 08017 Barcelona, Spain; 19 Infectious Diseases Unit, Department of Internal Medicine. Hospital de Barcelona, 08034 Barcelona, Spain; 20 Infectious Diseases Department, Hospital Universitari de Bellvitge, Institut d’Investigació Biomèdica de Bellvitge-IDIBELL, 08907 L’Hospitalet de Llobregat, Spain

**Keywords:** antibiotic stewardship, catheter-related bloodstream infection, coagulase-negative staphylococci, healthcare related infection

## Abstract

According to clinical guidelines, the management of catheter-related bloodstream infections (CRBSI) due to coagulase-negative staphylococci (CoNS) includes catheter removal and antibiotic treatment for 5 to 7 days. However, in low-risk episodes, it remains uncertain whether antibiotic therapy is necessary. This randomized clinical trial aims to determine whether the non-administration of antibiotic therapy is as safe and effective as the recommended strategy in low-risk episodes of CRBSI caused by CoNS. With this purpose, a randomized, open-label, multicenter, non-inferiority clinical trial was conducted in 14 Spanish hospitals from 1 July 2019 to 31 January 2022. Patients with low-risk CRBSI caused by CoNS were randomized 1:1 after catheter withdrawal to receive/not receive parenteral antibiotics with activity against the isolated strain. The primary endpoint was the presence of any complication related to bacteremia or to antibiotic therapy within 90 days of follow-up. The secondary endpoints were persistent bacteremia, septic embolism, time until microbiological cure, and time until the disappearance of a fever. EudraCT: 2017-003612-39 INF-BACT-2017. A total of 741 patients were assessed for eligibility. Of these, 27 were included in the study; 15 (55.6%) were randomized to the intervention arm (non-antibiotic administration) and 12 (44.4%) to the control arm (antibiotic therapy as per standard practice). The primary endpoint occurred in one of the 15 patients in the intervention group (septic thrombophlebitis) and in no patients in the control group. The median time until microbiological cure was 3 days (IQR 1–3) in the intervention arm and 1.25 days (IQR 0.5–2.62) in the control arm, while the median time until fever resolution was zero days in both arms. The study was stopped due to the insufficient number of recruited patients. These results seem to indicate that low-risk CRBSI caused by CoNS can be managed without antibiotic therapy after catheter removal; efficacy and safety are not affected.

## 1. Introduction

The use of endovascular catheters is a standard practice in the hospital setting [1]. A recent study reported that around 80% of patients admitted to medical wards have one or more catheters inserted during admission, of which 95% are short-term peripheral catheters [2]. Vascular catheters are among the most frequent causes of nosocomial bacteremia, being associated with between 15% and 30% of all cases [3,4]. The rate of catheter-related bloodstream infection (CRBSI) has been estimated between 0.1 and 2.7 episodes per 1000 catheter days [5], and *Staphylococcus epidermidis* has been identified as the most frequently involved microorganism [6,7,8].

The usual approach to CRBSI consists of catheter withdrawal and the administration of antibiotic treatment. The existence of local signs of infection is an absolute criterion for device removal. Recurrent bacteremia and metastatic infections are frequent. However, in certain circumstances, catheter retention may be considered, especially when the source of the infection is an indwelling permanent central venous catheter. Antibiotic treatment is also started and is usually prescribed for 5–14 days, depending on the causative microorganism [9,10,11].

CRBSI caused by coagulase-negative staphylococci (CoNS)—is a distinctive scenario because of the microorganism’s low virulence and the difficulty of differentiating between contamination and true bacteremia, given that CoNS are frequent skin colonizers. An exception is *Staphylococcus lugdunensis*, which is an infrequent but aggressive cause of infectious endocarditis [7,12].

Management of CoNS CRBSI includes catheter withdrawal and the administration of antibiotic treatment for 5–7 days (level of evidence B-III). On occasion, in accordance with expert opinion (level of evidence C-III), catheter withdrawal may be considered without the initiation of any specific treatment [9,13,14,15]. With cure rates around 100%, this strategy can be applied in immunocompetent patients who are hemodynamically stable and have no prosthetic materials other than the catheter [9]. However, to date, these two therapeutic options have not been compared in a randomized clinical trial. The aim of the present multicenter clinical trial is to determine whether the non-administration of antibiotics is as safe and effective as antibiotic therapy in CRBSI caused by CoNS among patients with low risk factors for complications managed with vascular catheter withdrawal.

## 2. Results

### 2.1. Study Population

**Figure 1:**
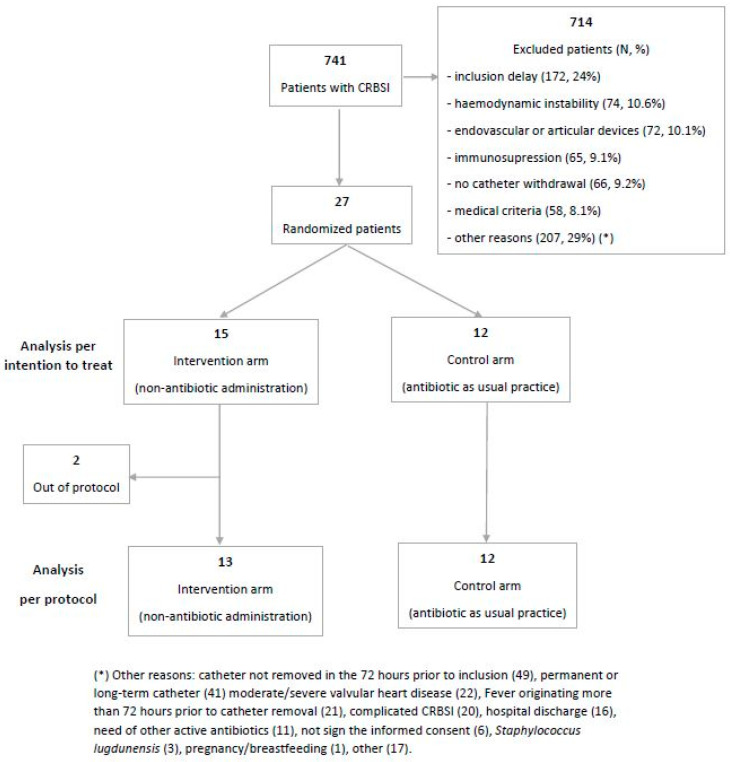
Patient flowchart.

A total of 741 patients had a CRBSI caused by CoNS during the study period. Of these, 27 were included in the study; 15 (55.6%) were randomized to the intervention arm (non-antibiotic administration) and 12 (44.4%) to the control arm (antibiotic as per standard practice) (Figure 1). Clinical and demographic characteristics are displayed in Table 1. The median age was 62 years, 52% were women, 55% had a Charlson score greater than 4, 11% had cirrhosis, 7% had chronic obstructive pulmonary disease and chronic renal failure, and 3.7% had heart failure. Most episodes occurred in surgical units (60%), with CVC (44%) and PICVC (40%) being the most frequently involved catheters. Seventy per cent of catheters were used for parenteral nutrition. At the moment of inclusion, 63% of patients had fever.

The study was stopped before achieving the expected sample as patient recruitment was too slow. At the end, 27 patients were randomized.

### 2.2. Primary Outcome

In the ITT analysis, the primary endpoint was recorded in one of the 15 patients in the intervention group (95%CI: 0.3–34), who presented septic thrombophlebitis, and in none of the patients in the control group (95%CI: 0–30.1) (Table 2). The per-protocol analysis presented the same results, with one of the 13 patients in the intervention group presenting the primary endpoint (with septic thrombophlebitis) (95%CI: 0.4–37.9), and none of the controls (Table 3) (95%CI: 0–30.1). Apart from septic trombophlebitis, no other adverse events were recorded.

### 2.3. Secondary Outcomes

In the ITT analysis, the median time until microbiological cure was 3 days (IQR 1–3) in the intervention arm and 1.25 (IQR 0.5–2.6) in the control arm. The median time until fever resolution was 0 days (IQR 0-0) in both arms (Table 4). In the per-protocol analysis, the median time until microbiological cure was 1.5 days (IQR 1–3) in the intervention arm and 1.25 (IQR 0.5–2.6) in the control arm. The median time until fever resolution was 0 days (0-0) in both arms (Table 5).

At Day 7 after inclusion, one death occurred in the intervention group. At Day 90, four deaths had occurred (one in the control group and three in the intervention group), and no CRBSI relapses were observed. No death was related to the CRBSI or the antibiotic treatment.

### 2.4. Screening Failure

A total of 714 patients were excluded (reasons for exclusion are summarized in Figure 1). Among them, 3.85% died in the first seven days after CRBSI; 90-day mortality was 18.3%. Hemodynamic instability was significantly associated with 7- and 90-day mortality [RR 1.23 (1.8–12.97) and 1.9 (1.11–2.97) respectively)]. Time until hospital discharge was 18 days (IQR 9–35). Hemodynamic instability and immunosuppression were associated with longer hospital admission after CRBSI.

## 3. Discussion

This is the first randomized clinical trial comparing two strategies against low-risk CRBSI caused by CoNS, in which patients were randomly assigned after catheter withdrawal to receive no antibiotic treatment (interventional arm) or intravenous antibiotic, as usual practice (control arm). After the inclusion of 27 patients, only one of the 15 patients included in the interventional group experienced a complication (septic thrombophlebitis) and no patient died due to the CoNS CRBSI. This strategy has the advantage of avoiding unnecessary antibiotic adverse effects and reducing broad-spectrum antibiotic use against the most frequent cause of nosocomial CRBSI. In addition, since most CoNS are resistant to beta-lactams, vancomycin is usually needed for its treatment. Thus, the selection pressures for vancomycin-resistant *Enterococcus* and vancomycin- intermediate *Staphylococcus aureus* may be reduced by decreasing the use of vancomycin.

Due to the low virulence of CoNS, these infections usually have benign courses. However, several risk factors for CoNS CRBSI-related mortality have been described, associated with either the host [16], the severity of bacteremia, or the maintenance of the catheter [17]. Other studies have observed an increased risk of recurrent bacteremia [18,19] or longer hospital stays [20,21] if the catheter is not removed.

Despite the low virulence of CoNS, the current guidelines recommend catheter withdrawal and 5–7 days of antibiotic therapy [9,13]. However, shortened antibiotic treatment, or even no antibiotic at all, has been proposed in patients without risk factors for complications [7,9]. In a recent retrospective study, San Juan et al. found no differences in mortality or recurrence risk when comparing short (≤3 days) and long (>3 days) antibiotic regimens in CRBSI caused by CoNS. However, differences in baseline characteristics of the two groups were observed, given that patients receiving long antibiotic therapy had more risk factors for complications and a more severe clinical presentation [19]. In another retrospective study, Hebesien et al. did not observe significant differences in short- and long-term complications between the group of patients receiving antibiotic treatment and those that did not. However, in that study, CRBSI was defined as the growth of CoNS in the tip culture and in one rather than two bloodstream cultures, and it was difficult to distinguish from colonization. In addition, in the antibiotic group, there was a significantly higher proportion of immunosuppressed patients [22]. Similarly, in another retrospective cohort study of risk factors for mortality in CoNS bacteremia, Park et al. did not observe significant differences between the groups managed with or without appropriate empirical treatment. Pitt bacteremia score and retention of eradicable foci were identified as risk factors for mortality [17].

The main limitation of our study is the scarce number of patients included, which did not allow for the achievement of the calculated sample size. Undoubtfully, it was due to the restrictive definition of low-risk bloodstream infection. In addition, the SARS-CoV-2 pandemic presented an additional difficulty for patient recruitment. In fact, 172 patients could not be included due to inclusion delay. However, no clinical trials have compared these strategies to date. Despite the failure to obtain the desired sample size, our results seem to indicate that low-risk CoNS CRBSI may be managed without the administration of antibiotics, making this strategy safe and effective.

## 4. Materials and Methods

### 4.1. Setting

The study was conducted at 14 Spanish National Health System hospitals that participate in the healthcare-related Infections Study Group of the Spanish Society of Infectious Diseases and Clinical Microbiology (https://geiras-seimc.org, accessed on 6 April 2023).

### 4.2. Study Design

Randomized, open-label, multicenter clinical trial to demonstrate the non-inferiority of non-administration of antibiotics compared to antibiotic treatment in episodes of low-risk CoNS CRBSI from 1 July 2019 to 31 January 2022.

Before randomization, patients were treated according to usual practice. Patients were randomly 1:1 assigned after catheter withdrawal to: (1) Intravenous antibiotic with activity against the isolated CoNS strain for 5–7 days, at the discretion of the clinician responsible; and (2) No antibiotic treatment after randomization. The treatment arm was assigned centrally and automatically by a computer platform once the patient’s eligibility criteria were verified. Randomization was stratified according to the participating center and the use of any antibiotic treatment at the time of enrollment.

Patients were monitored daily for at least seven days or until treatment was completed if CRBSI became complicated. Blood cultures were performed on Days 1 and 3 in all cases, and daily if previous blood cultures were positive or if fever or signs of sepsis persisted. Patients were followed up for 90 days after the diagnosis of the CRBSI caused by CoNS.

If viable bacteria were detected in blood cultures for more than 48 h after inclusion in the study, patients initiated antibiotic treatment, and the case was considered as a failure of the initial strategy.

The study was interrupted in the case of voluntary withdrawal of consent, protocol violation, serious adverse event, loss to follow-up, or at the discretion of the attending physician or study investigator.

### 4.3. Study Population

Patients ≥18 years with CRBSI caused by CoNS without risk factors for complications.

Inclusion criteria: age ≥ 18 years, CRBSI caused by CoNS (see definition), catheter removal in the 72 h prior to inclusion, provision of signed informed consent to participate in the study.

Exclusion criteria

- Pregnancy or breastfeeding
- Growth of CoNS of the species *Staphylococcus lugdunensis*
- Patients whose catheter had not been removed in the 72 h prior to inclusion
- Patients with moderate-to-severe valvular heart disease
- Patients with compromised immunity or neutropenia < 500 Ne/uL
- Hemodynamically unstable patients presenting as septic shock
- Patients with evidence of septic thrombophlebitis or distant infection
- Patients with artificial endovascular or articular devices
- Patients with permanent or long-term catheters
- Any circumstance that, at the discretion of the physician responsible, might entail a clinical risk, might negatively affect the patient’s participation in the study, or might interfere with the evaluations
- Fever originating more than 72 h prior to catheter removal.

### 4.4. Safety Data

All adverse events during the study were recorded, and those related to the infection or to the antibiotic administered were reported to the sponsor within 24 h of the diagnosis.

### 4.5. Definitions [23]

CRBSI caused by CoNS: Detection of CoNS growth (excluding *Staphylococcus lugdunensis*) in a patient with a vascular catheter, in at least two blood cultures obtained from peripheral blood in a patient with clinical manifestations of infection without other apparent sources of bacteremia, with the exception of the catheter itself and one of the following:

(1) Quantitative hemocultures with detection of the same microorganism, with a proportion of 5:1 or more between the blood obtained from the lumens of a central venous catheter (CVC) or a peripheral-inserted central catheter (PICC), and that obtained from a peripheral vein.
(2) Semi-quantitative culture (>15 UFC/catheter segment) or quantitative (>10^3^ UFC/catheter segment) with detection of the same microorganism (at species level with identical antibiogram) as in the hemocultures obtained from peripheral blood.
(3) Time to positivity of the hemocultures above two hours between the hemocultures obtained from blood from a peripheral vein and those obtained from the lumen of a venous catheter.
(4) Presence of inflammatory signs or of purulent secretion at the point of insertion or in the path of the subcutaneous tunnel of a venous catheter of any type.
(5) Resolution of the clinical signs and symptoms after the removal of a CVC or PICC or a suitable antibiotic treatment.
- Low-risk catheter-related bloodstream infection: CRBSI in a hemodynamically stable, immunocompetent and with lack of risk factors for local or distant complications.
- Complicated bacteremia: evidence of distant infection or septic thrombophlebitis.
- Persistent bacteremia: detection of CoNS growth 48 h after randomization.
- Microbiological cure: lack of growth in blood cultures.

Time until microbiological cure: days from randomization until the first negative blood culture

Time until fever resolution: days with fever from randomization.

### 4.6. Endpoints

The primary endpoint was the presence of one of the following: persistent bacteremia, complicated bacteremia, or the presence of any complication related to the administration of antibiotics during the study follow-up.

The following secondary endpoints were assessed separately: persistent bacteremia, complicated bacteremia, time until microbiological cure, and time until fever resolution.

### 4.7. Sample Size

It was expected that the rates of patients presenting the composite variable would be 12% for the group of patients treated with antibiotics and 7% for the group without antibiotics. Considering a delta of 7%, a sample size of 125 patients per group would allow us to detect non-inferiority with a beta error of 90% and a one-sided alpha error of 2.5%.

### 4.8. Microbiology Studies

Two sets of two blood samples obtained from a peripheral vein were obtained from all patients with a suspected bloodstream infection. An additional blood sample was also obtained through the catheter. When possible, the catheter tip was cultured after removal. Each hospital identified the strain and performed preliminary susceptibility tests. Blood samples were processed at the microbiology laboratories of each participating center in accordance with standard operating procedures. CoNS identification was carried out through matrix-assisted laser desorption ionization time-of-flight mass spectrometry (MALDI-TOF MS, Bruker Daltonik GmbH, Bremen, Germany). Susceptibility to antibiotics was assessed by the microdilution method and interpreted according to the European Committee for Antimicrobial Susceptibility Testing (EUCAST) recommendations and criteria [24].

### 4.9. Statistical Analysis

The statistical analysis was carried out in accordance with the principles specified in the International Conference on Harmonization (ICH) Topic E9 (CPMP/ICH/363/96) [25]. The general approach to the statistical analysis comprised:

Descriptive statistical methods, including number of missing values, number of valid values, their observed range, mean, median, standard deviation, and 95% confidence interval for the mean were used to describe the quantitative variables. Categorical variables were described as the number of cases and the percentage with respect to the total. The level of statistical significance was set at a *p*-value ≤ 0.05. Main variable analysis was carried out by calculating the percentage accompanied by a 95% confidence interval for each group, and a z-test was used to test the hypothesis. Secondary variables were compared using the general strategies described previously, in addition to the relative risk accompanied by a 95% confidence interval.

Statistical analyses were performed with R software version 4.2.1 or higher.

Two different analyses were performed: the intention to treat analysis (ITT), which included all randomized patients in the study, ignoring protocol deviations, and the per-protocol (PP) analysis, with the inclusion of a subset of the ITT patients who completed the study without any major protocol violations.

## Figures and Tables

**Table 1:** Clinical and demographic characteristics of 27 patients included in the study.

	Intervention (%) *N* = 15	Control (%) *N* = 12
**Patient characteristics**		
Age, mean	64	59
Female sex	8 (53.3)	6 (50)
Heart failure	1 (7.1)	0
Cirrhosis	2 (14.3)	1 (8.3)
Chronic obstructive pulmonary disease	1 (7.1)	1 (8.3)
Renal chronic failure	2 (14.3)	0
Charlson ≥ 4	12 (80)	3 (25)
**Clinical presentation**		
Sepsis	1 (6.7)	1 (8.3)
Fever	7 (46.7)	10 (83.3)
Local infection	5 (33.3)	3 (25)
**Catheter characteristics**		
Days since insertion (median, IQR)	11 days (7–15)	11 days (8.7–14.2)
Site of acquisition		
Medical ward	5 (33.3)	6 (50)
Surgical ward	10 (66.7)	6 (50)
Catheter type		
Midline	1 (6.7)	0 (0)
CVC	7 (46.7)	5 (41.7)
PICVC	5 (33.3)	6 (50)
PVC	2 (13.3)	1 (8.3)
Site of insertion		
Jugular	6 (40)	2 (16.7)
Subclavian	1 (6.7)	2 (16.7)
Basilic/cephalic vein	6 (40)	6 (50)
Radial	2 (13.3)	2 (16.7)
Use		
- parenteral nutrition	10 (66.7)	9 (75)
- medication or serums	5 (33.3)	3 (25)

CVC: central venous catheter, PICVC: peripheral inserted central venous catheter; PVC: peripheral venous catheter.

**Table 2:** Intention to treat analysis.

Group	Failure	*n*	N (Total)	%	CI [95%]
**Control**	No	12	12	100.0	[69.9–100]
**Control**	**Yes**	0	12	0.0	[0–30.1]
**Intervention**	No	14	15	93.3	[66–99.7]
**Intervention**	**Yes**	1	15	6.7	[0.3–34]

**Table 3:** Per-protocol analysis.

Group	Failure	*n*	N (Total)	%	CI [95%]
**Control**	No	12	12	100.0	[69.9–100]
**Control**	**Yes**	0	12	0.0	[0–30.1]
**Intervention**	No	12	13	92.3	[62.1–99.6]
**Intervention**	**Yes**	1	13	7.7	[0.4–37.9]

**Table 4:** Secondary outcomes: relative risk per group, per intention to treat.

	Control Group			Intervention Group					
Secondary Variables	*n*	(%)	CI 95%	*n*	(%)	CI 95%	RR	CI 95%	*p*
Persistent bacteraemia	0	0.00	[0–30.13]	1	6.67	[0.35–33.97]	.	.	.
Complicated bacteraemia	0	0.00	[0–30.13]	1	6.67	[0.35–33.97]	.	.	.
Treatment side-effect	0	0.00	[0–30.13]	0	0	[0–25.35]	.	.	.
7-day mortality	0	0.00	[0–30.13]	1	6.67	[0.35–33.97]	.	.	.
90-day mortality	1	8.33	[0.44–40.25]	3	20.00	[5.31–48.63]	2.4	[0.28–20.24]	0.42
Time until microbiological cure (median, IQR)	1.25 days		[0.5; 2.62]	3 days		[1; 3]			
Time until fever extinction (median, IQR)	0 days		[0; 0]	0 days		[0; 0]			

**Table 5:** Secondary outcomes: relative risk per group, per protocol.

	Control Group			Intervention Group					
Secondary Variables	*n*	(%)	CI 95%	*n*	(%)	CI 95%	RR	CI 95%	*p*
Persistent bacteraemia	0	0.00	[0–30.13]	1	7.69	[0.4–37.91]	.	.	.
Complicated bacteraemia	0	0.00	[0–30.13]	1	7.69	[0.4–37.91]	.	.	.
Treatment side-effect	0	0.00	[0–30.13]	0	0	[0–28.34]	.	.	.
7-day mortality	0	0.00	[0–30.13]	1	7.69	[0.4–37.91]	.	.	.
90-day mortality	1	8.33	[0.44–40.25]	3	23.08	[6.16–54.02]	2.77	[0.33–23.14]	0.34
Time until microbiological cure (median, IQR)	1.25 days		[0.5; 2.62]	1.5 days		[1; 3]			
Time until fever extinction (median, IQR)	0 days		[0; 0]	0 days		[0; 0]			

## Data Availability

Not applicable.

## References

[1] Pérez-Granda M.J., Guembe M., Rincón C., Muñoz P., Bouza E. (2014). A prevalence survey of intravascular catheter use in a general hospital. J. Vasc. Access.

[2] Guembe M., Pérez-Granda M.J., Capdevila J.A., Barberán J., Pinilla B., Martín-Rabadán P., Bouza E., Millán J., Pérez de Oteyza C., Muiño A. (2017). Nationwide study on peripheral-venous-catheter-associated-bloodstream infections in internal medicine departments. J. Hosp. Infect..

[3] Wisplinghoff H., Bischoff T., Tallent S.M., Seifert H., Wenzel R.P., Edmond M.B. (2004). Nosocomial bloodstream infections in US hospitals: Analysis of 24,179 cases from a prospective nationwide surveillance study. Clin. Infect. Dis..

[4] Zarb P., Coignard B., Griskeviciene J., Muller A., Vankerckhoven V., Weist K., Goossens M.M., Vaerenberg S., Hopkins S., Catry B. (2012). The european centre for disease prevention and control (ECDC) pilot point prevalence survey of healthcare-associated infections and antimicrobial use. Eurosurveillance.

[5] Maki D.G., Kluger D.M., Crnich C.J. (2006). The risk of bloodstream infection in adults with different intravascular devices: A systematic review of 200 published prospective studies. Mayo Clin. Proc..

[6] Weiner L.M., Webb A.K., Limbago B., Dudeck M.A., Patel J., Kallen A.J., Edwards J.R., Sievert D.M. (2016). Antimicrobial-Resistant Pathogens Associated With Healthcare-Associated Infections: Summary of Data Reported to the National Healthcare Safety Network at the Centers for Disease Control and Prevention, 2011–2014. Infect. Control Hosp. Epidemiol..

[7] Becker K., Heilmann C., Peters G. (2014). Coagulase-negative staphylococci. Clin. Microbiol. Rev..

[8] Badia-Cebada L., Peñafiel J., Patrick P., Andrés M., Càmara J., Domenech D., Jiménez-Martínez E., Marrón A., Moreno E., Pomar V. (2022). Trends in the Epidemiology of Catheter-Related Bloodstream Infections Towards a Paradigm Shift. Eurosurveillance.

[9] Mermel L.A., Allon M., Bouza E., Craven D.E., Flynn P., O’Grady N.P., Raad I.I., Rijnders B.J.A., Sherertz R.J., Warren D.K. (2009). Clinical practice guidelines for the diagnosis and management of intravascular catheter-related infection: 2009 update by the infectious diseases society of America. Clin. Infect. Dis..

[10] Lebeaux D., Fernández-Hidalgo N., Chauhan A., Lee S., Ghigo J.M., Almirante B., Beloin C. (2014). Management of infections related to totally implantable venous-access ports: Challenges and perspectives. Lancet Infect. Dis..

[11] Raad I., Hanna H., Maki D. (2007). Intravascular catheter-related infections: Advances in diagnosis, prevention, and management. Lancet Infect. Dis..

[12] O’Grady N.P., Alexander M., Burns L.A., Dellinger E.P., Garland J., Heard S.O., Lipsett P.A., Masur H., Mermel L.A., Pearson M.L. (2011). Guidelines for the prevention of intravascular catheter-related infections. Am. J. Infect. Control.

[13] Chaves F., Garnacho-Montero J., del Pozo J.L., Bouza E., Capdevila J.A., de Cueto M., Domínguez M.Á., Esteban J., Fernández-Hidalgo N., Fernández Sampedro M. (2018). Executive summary: Diagnosis and Treatment of Catheter-Related Bloodstream Infection: Clinical Guidelines of the Spanish Society of Clinical Microbiology and Infectious Diseases (SEIMC) and the Spanish Society of Intensive Care Medicine and Coronary Units (SEMICYUC). Enferm. Infecc. Microbiol. Clin..

[14] Lutwick L., Al-Maani A.S., Mehtar S., Memish Z., Rosenthal V.D., Dramowski A., Lui G., Osman T., Bulabula A., Bearman G. (2019). Managing and preventing vascular catheter infections: A position paper of the international society for infectious diseases. Int. J. Infect. Dis..

[15] Timsit J.F., Baleine J., Bernard L., Calvino-Gunther S., Darmon M., Dellamonica J., Desruennes E., Leone M., Lepape A., Leroy O. (2020). Expert consensus-based clinical practice guidelines management of intravascular catheters in the intensive care unit. Ann. Intensive Care.

[16] Cui J., Liang Z., Mo Z., Zhang J. (2019). The species distribution, antimicrobial resistance and risk factors for poor outcome of coagulase-negative staphylococci bacteraemia in China. Antimicrob. Resist. Infect. Control.

[17] Park S.Y., Kwon K.H., Chung J.W., Huh H.J., Chae S.L. (2015). Coagulase-negative staphylococcal bacteremia: Risk factors for mortality and impact of initial appropriate antimicrobial therapy on outcome. Eur. J. Clin. Microbiol. Infect. Dis..

[18] Raad I., Kassar R., Ghannam D., Chaftari A.M., Hachem R., Jiang Y. (2009). Management of the catheter in documented catheter-related coagulase-negative staphylococcal bacteremia: Remove or retain?. Clin. Infect. Dis..

[19] San-Juan R., Martínez-Redondo I., Fernández-Ruiz M., Ruiz-Ruigómez M., Corbella L., Hernández-Jiménez P., Silva J.T., López-Medrano F., Recio R., Orellana M.Á. (2019). A short course of antibiotic treatment is safe after catheter withdrawal in catheter-related bloodstream infections due to coagulase-negative staphylococci. Eur. J. Clin. Microbiol. Infect. Dis..

[20] Olaechea P.M., Álvarez-Lerma F., Palomar M., Insausti J., López-Pueyo M.J., Martínez-Pellús A., Cantón M.L. (2011). Impacto de la bacteriemia primaria y relacionada con catéterintravascular causadapor Staphylococcus coagulasa negativo enpacientes críticos. Med. Intensiv..

[21] Molina J., Peñuela I., Lepe J.A., Gutiérrez-Pizarraya A., Gómez M.J., García-Cabrera E., Cordero E., Aznar J., Pachón J. (2013). Mortality and hospital stay related to coagulase-negative Staphylococci bacteremia in non-critical patients. J. Infect..

[22] Hebeisen U.P., Atkinson A., Marschall J., Buetti N. (2019). Catheter-related bloodstream infections with coagulase-negative staphylococci: Are antibiotics necessary if the catheter is removed?. Antimicrob. Resist. Infect. Control.

[23] Nosocomial Infection Surveillance Programme at Catalan Hospitals (VINCat). 2015 Manual [Internet]. https://catsalut.gencat.cat/web/.content/minisite/vincat/documents/manuals/Manual-VINCat-2015-english.pdf.

[24] EUCAST European Committee on Antimicrobial Susceptibility Testing Breakpoint tables for interpretation of MICs and zone diameters Version 10.0. https://www.eucast.org/fileadmin/src/media/PDFs/EUCAST_files/Breakpoint_tables/v_10.0_Breakpoint_Tables.pdf.

[25] ICH E9 Expert Working Group (1999). Statistical principles for clinical trials (ICH Harmonized tripartite guideline E9). Stat. Med..

